# Rational design of artificial redox-mediating systems toward upgrading photobioelectrocatalysis

**DOI:** 10.1007/s43630-021-00099-7

**Published:** 2021-09-22

**Authors:** N. Samali Weliwatte, Matteo Grattieri, Shelley D. Minteer

**Affiliations:** 1 Department of Chemistry, University of Utah, Salt Lake City, UT 84112 USA; 2 Dipartimento Di Chimica, Università Degli Studi Di Bari “Aldo Moro”, Via E. Orabona 4, 70125 Bari, Italy; 3 IPCF-CNR Istituto Per I Processi Chimico Fisici, Consiglio Nazionale Delle Ricerche, Via E. Orabona 4, 70125 Bari, Italy

**Keywords:** Photobioelectrochemical cells, Biohybrid, Electrical wiring, Diffusible redox mediators, Redox polymers

## Abstract

Photobioelectrocatalysis has recently attracted particular research interest owing to the possibility to achieve sunlight-driven biosynthesis, biosensing, power generation, and other niche applications. However, physiological incompatibilities between biohybrid components lead to poor electrical contact at the biotic-biotic and biotic-abiotic interfaces. Establishing an electrochemical communication between these different interfaces, particularly the biocatalyst-electrode interface, is critical for the performance of the photobioelectrocatalytic system. While different artificial redox mediating approaches spanning across interdisciplinary research fields have been developed in order to electrically wire biohybrid components during bioelectrocatalysis, a systematic understanding on physicochemical modulation of artificial redox mediators is further required. Herein, we review and discuss the use of diffusible redox mediators and redox polymer-based approaches in artificial redox-mediating systems, with a focus on photobioelectrocatalysis. The future possibilities of artificial redox mediator system designs are also discussed within the purview of present needs and existing research breadth.

## Introduction

The exponential global energy demand exceeded 400 EJ (EJ = 10^18^) per year in 2020 due to growing anthropogenic activity [1], and despite the populational and economic setbacks pertaining to the COVID-19 pandemic, global electricity demand is forecasted to increase by 3% in 2021 [2]. Traditional fossil fuel energy (i.e., coal, natural gas, oil) primarily caters to this demand [3, 4], with concomitant harmful carbon and greenhouse gas emissions exacerbating global climate change [5]. Therefore, a carbon–neutral, circular energy economy has become an increasingly desirable goal. In this context, solar energy holds much promise among renewable sources, being green, sustainable and available on earth in quantities surpassing the current human energy consumption requirements by 10^6^-fold [2, 6, 7]. Significant advances in solid-state photovoltaics are being achieved to harness solar energy into more pliable forms, resulting in solar cells with efficiencies approaching 20% [8–11]. Additional solar-harnessing technologies are required to complement the current solar energy landscape in order to potentially address existing limitations. Thereon, “artificial photosynthesis” is an alternative route that utilizes artificial, tunable, selective, and efficient photocatalysts, which emulates natural photosynthetic units in order to harvest solar energy for substrates-synthesis and power generation [12–15]. Some restrictions in artificial photosynthesis are the limited long-term stability, and the need for high purity, heterogenization of catalysts for efficient product isolation, stringent catalytic conditions, and in the case of certain catalysts, the presence of noble metal centers [15].

**Fig. 1:**
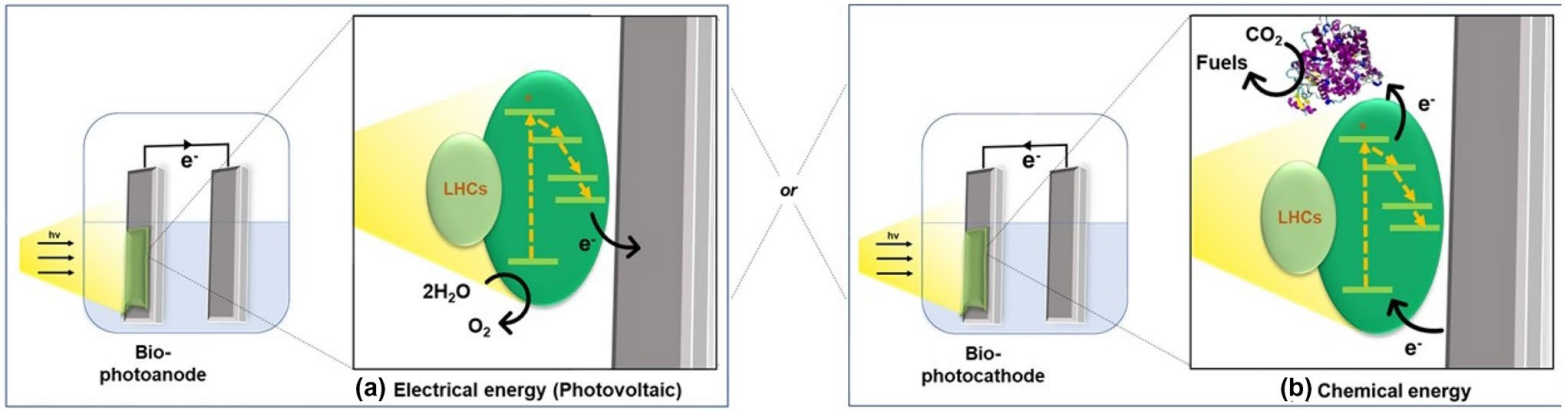
Schematic representation of the photobioelectrocatalytic mechanism; generation of charge separations in photosystems by harvesting light and the consequent conversion of that energy to **a** electrical energy or, **b** chemical energy, via redox catalysis

Photobioelectrocatalysis is a semi-artificial photosynthetic technique that harnesses solar energy utilizing natural photosynthetic units, which have specifically evolved over billions of years to convert solar energy into electrical and/or chemical energy (Fig. 1) [6, 16]. Solar energy is absorbed by specialized light harvesting centers (LHCs) in these photosynthetic entities, with quantum efficiencies approaching 100%. The resulting high energy electron fluxes in LHCs traverse through what has been commonly defined as the “Z-scheme” during oxygenic photosynthesis [7, 17, 18]. In biophotovoltaic technologies, the traversing high energy electron flux is accepted by the electrode in contact with the photosynthetic entity, generating a current output (Fig. 1a). Corresponding electron holes are replenished by the oxidation of water or an organic substrate, depending on the utilized photosynthetic entity. Alternatively, the high energy electron flux can be intercepted by a substrate that can be reduced generating chemical energy (e.g., CO_2_ reduction to form carbon-based fuels as feedstock chemicals, or N_2_ reduction) (Fig. 1b). The resulting electron holes at the photosynthetic entity are replenished by “oxidizing” an abiotic electrode. Biological entities, especially certain enzymes, have the sophistication to form complex carbon-based chemicals at this juncture that are not yet realized by artificial synthetic pathways under mild conditions [16, 19–21]. Therefore, photobioelectrocatalytic cell configurations now also include biotic components acting as light harvester and the biotic unit as the catalysts. It should be noted that in nature, crop plants and microalgae perform solar-to-biomass conversions respectively at under 1% and 7% efficiencies [19]. With these aspects in mind, photobioelectrocatalysis can amalgamate the selectivity of the natural phenomenon with the energy efficiency of the artificial counterpart. The resulting biohybrid systems must be further tuned in terms of catalytic site density, solar absorption, product yields, faradaic efficiency, long-term stability, and scalability to optimize photobioelectrocatalytic performance and applicational value [6, 16]. Therefore, photobioelectrocatalysis is a burgeoningly rich field of multidisciplinary science.

Photosynthetic entities utilized in this context can be broadly classified into four groups; (i) intact chloroplasts, (ii) microbial cells, (iii) isolated photosystems, and (iv) other enzymes. Photosynthetic microbial fuel cells constitute phototrophic microbes that either generate the high energy electron flux for reducing power, or provide sustenance to heterotrophic microbes that generate the reducing power [22]. More recently, a third type of fuel cell has been introduced, where a non-photosynthetic biological entity hybridizes with a photoactive artificial element in order to harvest solar energy. Overall, the abundance, relatively low cost, low to none-toxicity, good recyclability, and easy processing conditions of biological entities facilitate the broad accessibility of photobioelectrocatalysis [6, 16]. While large scale photobioelectrocatalytic applications may not be fathomable in the near future, there is much promise in niche, low-power applications, such as environmental biosensing and bio-electrosynthesis. In this Review, focus is posed on the omnipresent challenge encountered in photobioelectrocatalysis: the poor electrochemical communication at the biotic-abiotic interface predominantly due to the presence of (various layers of) insulating membranes.

## Improving the electric contact at the photosynthetic entity-electrode interface

Overarchingly, the membranous nature of photosynthetic entities impairs the solar energy conversion efficiencies of current photobioelectrocatalytic systems. The photosynthetic machinery in oxygenic organisms occurs in the inner membrane matrix called the thylakoid, which embeds bacterial reaction centers of transmembrane pigment-protein complexes in cyanobacteria, or constitutes tandem protein photosystems I and II in other photosynthetic species. The thylakoid lipid bilayer has a uniquely higher composition of galactosyl diglycerides ranging around 70–80%, compared to other bilayer membranes [23]. These galactolipids include, but are not limited to unevenly distributed monogalactosyl diacylglycerol, digalactosyl diglycerol, phosphatidylglycerol and sulfoquinovosyl diacylglycerol [24]. Cyanobacterial thylakoids are cytosolic, detached from the plasma membrane, and encapsulated by a peptidoglycan-based cell wall [25–27]. In algae and plants, thylakoids are compartmentalized in chloroplasts via inner and outer membranes, a periplasm, and a cell wall made of polysaccharides and glycoproteins, or solely polysaccharides. Chloroplast membranes are more resonant with the plasma membrane composition, and the carboxylic groups in their membrane proteins render a negative surface charge on chloroplasts under physiological conditions [28]. Conversely to oxygenic bacteria, in anoxygenic photosynthetic bacteria, the photosynthetic apparatus is located in the inner membrane. Irrespectively, for all photosynthetic organisms, membrane structures imbibe photosynthetic centers, provide photo- and metabolic stability, facilitate communication and exchange mechanisms vital for the sustenance of the entities.

**Fig. 2:**
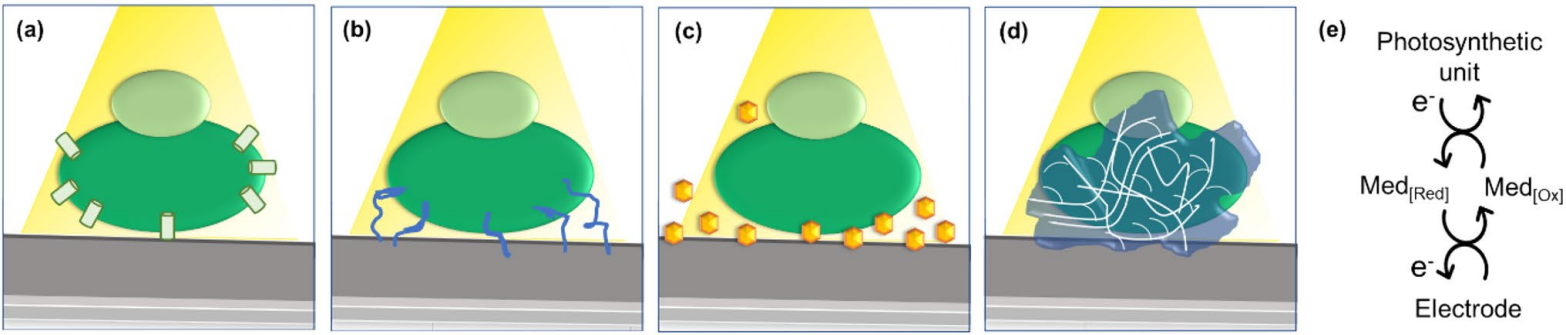
Schematic representations of EET; **a** E-DET via membrane proteins, **b** E-DET via intrinsic conductive molecular wires (e.g., pili, filaments), **c** E-MET via endogenous or exogenous diffusible redox mediators, **d** E-MET via redox polymers/matrices, **e** electron exchange during E-MET

However, during photobioelectrocatalysis, there is a certain degree of physiological incompatibility between these outer membranes and electrode surfaces. For instance, the chiefly protein- and lipid-based membranes are electrically insulating, barring extracellular electron transfer (EET) via direct- (E-DET) and mediated- (E-MET) electron transfer methods. E-DET typically occurs though electron-conducting membrane proteins (Fig. 2.(a)), or inherent physiological extensions seen in certain microbes (e.g., pili, nanowires, appendages, conductive matrices, etc.) (Fig. 2b) [29–32]. In addition, endogenous diffusible redox mediators such as plastoquinone, plastocyanin, cytochrome b_6_f, flavin, phenazine (Fig. 2c) naturally facilitate extracellular electron transfer. In the case of microbes that photosynthesize to accumulate biomass, E-DET routes are believed to be a means of dissipating oxidative stress by excessive photoelectron flux as a self-protecting mechanism, or a means of extracellular signaling between microbes, or a means of upholding bioavailability [22, 33]. Therefore, those pathways are not naturally-designed to channel their photoinduced reducing energy into artificially integrated components in an electrochemical set up (e.g., electrodes). The multiple encapsulating layers of membrane and cell walls between photosynthetic reactive centers and the electrode limit direct contact between the two. Outer sphere electron transfer between these sporadic electron conducting pathways or redox mediators and electrode surfaces at significant rates require close proximity between the said units according to the Marcus theory [19, 34, 35]. Membrane-less entities like isolated photosynthetic protein complex photosystem II (PSII) have their reactive centers embedded either sporadically or deeply enough to require specific orientations and secure deposition on the electrode surface in order to facilitate fast electron transfer. Self-aggregation of photosynthetic units and poor dispersion on the electrode surface also hinder fast electron transfer kinetics. Consequently, the photo-induced high energy electron flux is partially dissipated in extraneous metabolic activity reducing the capacity to generate electrical or chemical energy. Collectively, these complications are known as poor electrical- ‘contact’ or ‘wiring’ between photosynthetic entities and electrode surfaces, which necessitate additional electron transfer routes to efficiently pass on the reducing energy [36–39]. The future scale-up of photobioelectrocatalysis relies heavily on improving this electric contact at the biohybrid interface. For example, the theoretical current capacity of biophotovoltaic cells containing cyanobacteria is postulated to range in the orders of 0.07–0.77 mW cm^−2^ whereas their empirical values typically average in the 0.0001–0.01 mW cm^−2^ range [6, 40]. Technological advances in terms of improving anode design and cell architecture to implement better photosynthetic entity binding, film loading, increased electroactive surface area, mass transport capabilities and light transmittance have been reported [19, 41–43]. On the other hand, redox mediation for long-range EET in semiartificial photosynthetic systems (E-MET), can be broadly classified into two, namely; exogenous diffusible redox mediators (Fig. 2c) and redox polymers (Fig. 2d). Overarchingly, redox mediators and redox polymers are species that can undergo reversible or quasi-reversible electron transfers to repeatedly extract electron fluxes from photosynthetic reaction centers and channel them to electrodes (or vice versa) (Fig. 2e). Furthermore, the function of redox mediators has extended to biotic-biotic interfaces as well. This Review will focus on E-MET via diffusible and immobilized exogenous redox species in the context of photobioelectrocatalysis.

## Exogenous E-MET via diffusible redox mediators

**Fig. 3:**
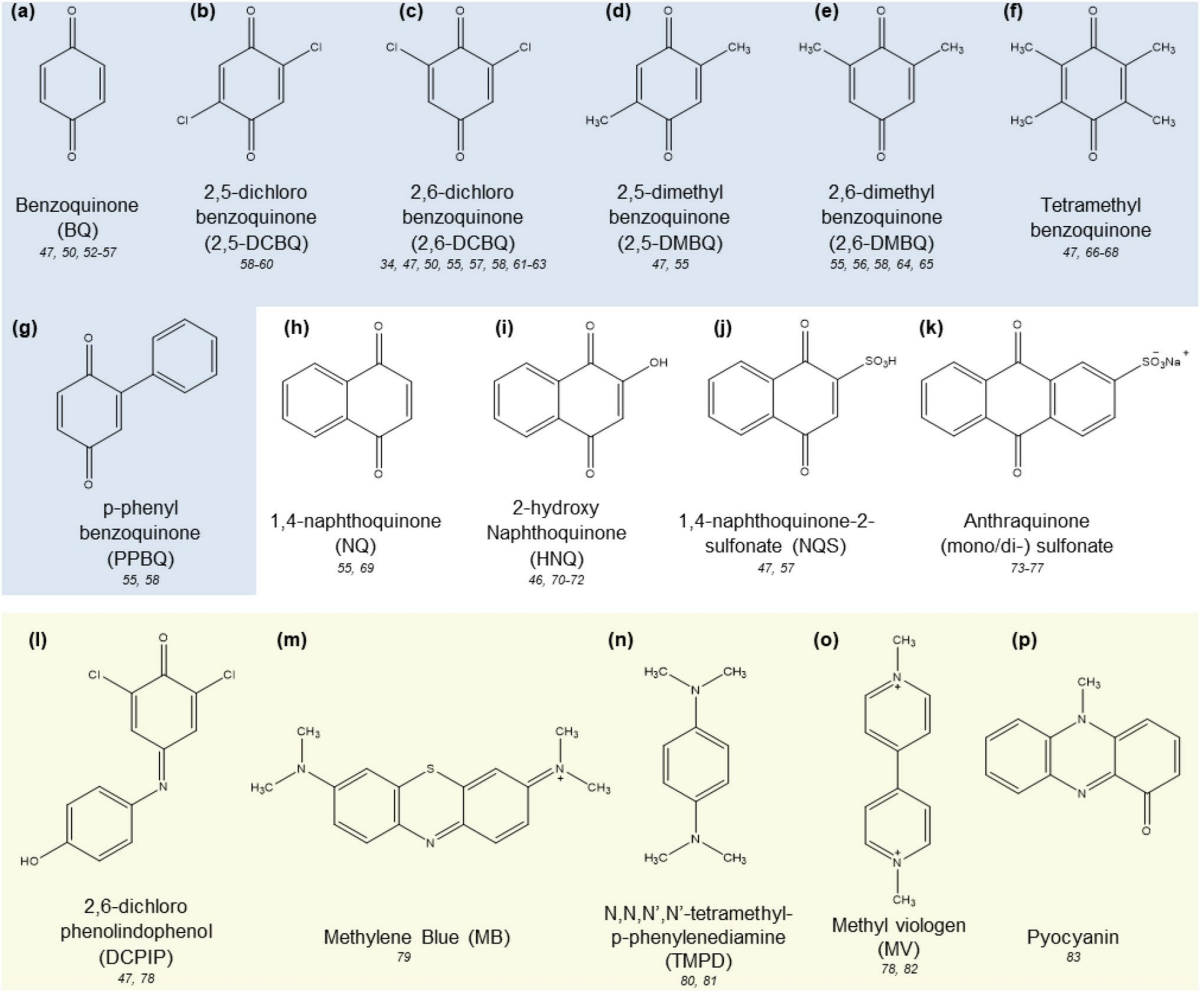
Common diffusible redox mediators; **a–g** benzoquinone derivatives, **h–k** benzoquinone derivatives with fused aromatic centers, **l–p** redox dye species

Diffusible redox mediators are either completely organic molecules, inorganic coordination complexes or organometallic compounds with ligand attachments that can undergo reversible redox reactions. Their functional edge lies in the anticipated ability to diffuse bidirectionally (‘*shuttle*’) across membranes and interstitial spaces. The use of such redox shuttles to improve catalytic performance has been reported since the early 1980s [22, 44]. Several common diffusible redox mediators are illustrated under the classification of benzoquinone (BQ) derivatives, BQ derivatives with fused aromatic rings, and redox dyes (Fig. 3).

### Benzoquinones

In 1985, Tanaka et al. reported one of the first microbial biophotovoltaic systems, constituting the cyanobacterium *Anabaena variabilis* M-2 and exogenous diffusible redox mediator 2-hydroxy-1,4-naphthoquinone (HNQ). (Fig. 3i) [45, 46]. The 49% coulombic efficiency increment upon illuminating the system was attributed to the HNQ interfacing the electrode and microbes. Generally, BQ derivatives are a popular class of diffusible redox mediators that can be considered biomimetic due to their structural resemblance to endogenous counterparts in biological systems such as plastoquinone, plastocyanin, menaquinone, and ubiquinone [47–49]. Hasan et al. investigated the suitability of nine BQs as redox mediators for thylakoid membranes on gold electrodes in phosphate buffer (Fig. 3a, c, d, f, j, l) [47]. It was shown that the chemical structures and properties such as the redox potentials, solubility, and the structural affinity of the BQs influenced the achievable photocurrent density [47, 48, 50]. Inductively electron withdrawing effect of Cl and Br in halogenated BQs results in higher redox potentials as opposed to BQs with inductively electron donating CH_3_ or mesomeric effect of OCH_3_. While methyl-substituted BQs have low affinity to the electron carrier Q_b_ pocket (the redox active site in the photosynthetic electron transport chain) in thylakoids, tetra-halogenated BQs have reported very high affinity. However, tetra- compared to di- halogenated BQs and dichlorophenol indophenol (DCPIP), naphthoquinone with extended π-systems showed lower photocurrent densities due to poor solubility in aqueous media. The study also investigated the influence of mediator concentration, applied potential, light intensity, and chlorophyll concentration on the photobioelectrocatalytic performance. Under optimized conditions, the system containing 2,6-dichloro-1,4-benzoquinone (DCBQ) yielded a 130 µA cm^−2^ current density. Some of these observations are parallel with the findings of Satoh et al., who investigated BQs and PS II under similar conditions [51].

The structure of the redox mediator is also found to be significant in terms of facilitating conductive chemical and steric interactions at the biotic-abiotic interface. For instance, Kato et al. reported that 1,4-naphthoquinone-2-sulfonate (NQS) was a better diffusible mediator compared to naphthoquinone with indium tin oxide (ITO)-PSII biohybrids, due to the former’s ability to create electrostatic interactions with the positively charged ITO (Fig. 3j) [52]. Further, BQs have been frequently utilized as redox mediators in photobioelectrocatalysis in conjunction with photosynthetic entities such as intact cyanobacteria *Synechococcus* sp. PPC7942 [53], *Thermosynechococcus elongatus * [54], PSII of *Thermosynechococcus elongatus * [52, 55], PSI of spinach [17], protoplast of marine algae *Bryopsis plumose * [56], etc.

Considerable research efforts have been aimed to expand the mechanistic understanding of redox mediation by BQs. Grattieri et al. screened seven BQ derivatives in order to wire the *Rhodobacter capsulatus*-Toray carbon electrode surface in a biophotovoltaic system [50]. The empirical data obtained with the BQ derivatives; p-benzoquione (Fig. 3a), 2-chloro-1,4-benzoquinone, 2,6-dichloro-1,4-benzoquinone (Fig. 3c), 2,3,5,-tetrachloro-1,4-benzoquinone, 2,3,5,6-tetrafluoro-1,4-benzoquinone, 2,3,5,6-tetrabromo-1,4-benzoquinone, and menadione were correlated to density functional theory based calculations, unveiling that the rate-limiting electron transfers occur at the lipophilic bacterial membranes, corresponding to a proton-decoupled single electron transfer process. Kasuno et al. calculated the photoinduced electron transfer rates from the purple bacteria *Rhodobacter sphaereoides* to DCBQ in biophotovoltaic systems using Michaelis–Menten kinetics [57]. Longatte et al. developed a “derivation parameter” (D) based on fluorescence measurements to quantify the electron extraction efficiency of seven BQ redox mediator species from genetically-engineered intact *Chlamydomonas reinhardtii * [58–60]. In 2018, the same group conducted a study on the stability of biophotovoltaic cells in terms of photocurrent density, utilizing planktonic *Chlamydomonas reinhardtii* systems respectively in conjunction with 2,6-DCBQ, 2,5-DCBQ, p-phenylbenzoquinone (PPBQ), 2,6-dimethylbenzoquinone (2,6-DMBQ) (Fig. 3b, c, e, g) [58]. The observed decreasing photocurrent density with consistent photo pulses was postulated to be due to either the photoinactivation of *Chlamydomonas reinhardtii* by the increasing photo flux [61], or the generation of a kinetic quencher by the BQs, or a direct deleterious effect by BQs interacting with cellular proteins, lipids, and DNA [62, 63]. The predominantly contributing mechanism of the three was shown to be determinant on auxiliary factors such as the chemical structure of the quinones, incubation time, light intensity, and illumination time. The empirical kinetic quenching model of quinones put forth by the study addresses a hitherto uninvestigated aspect of quinone-based redox mediation.

### Redox dyes

Herein, diffusible redox mediators that have distinct colors at specific redox states (*redox indicators*), and redox-active molecules with merely a characteristic color, are both classified as redox dyes. Several prominent redox dyes that have been explored in the photobioelectrocatalysis context include DCPIP, methylene blue (MB), and N,N,N’,N’-tetramethyl-p-phenylenediamine (TMPD), methyl viologen (MV) (Fig. 3l–p), and phenazines [17, 64].

#### DCPIP

**Fig. 4:**
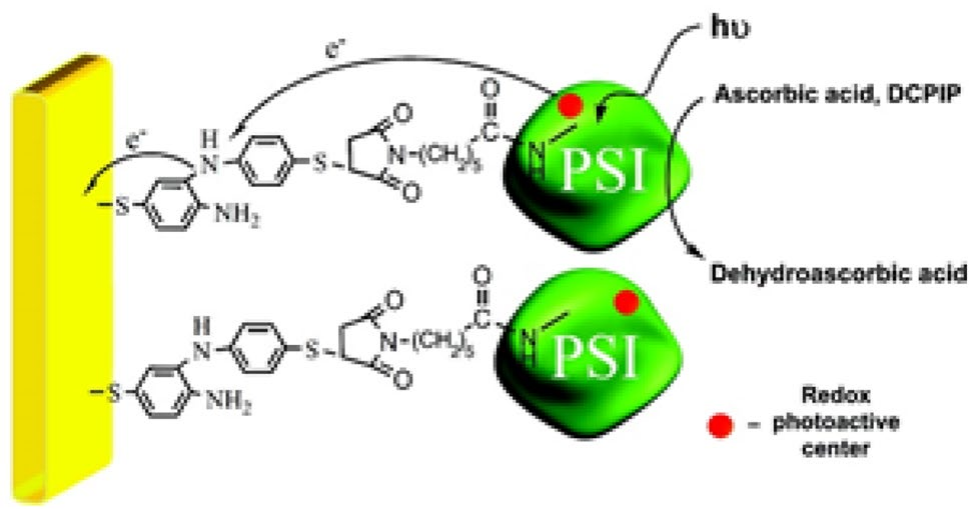
Schematic representation of a PSI-Au electrode biohybrid, where the PSI forms a monolayer on the Au surface by the bis-aniline cross-linkage. The crosslinks provide the electric contact at the biotic-abiotic interface, while ascorbic acid and DCPIP respectively function as electron donor and internal redox mediator. Adapted with permission from O. Yehezkeli, O. Wilner, R. Tel-Vered, D. Roizman-Sade, R. Nechushtai, and I. Willner. Generation of Photocurrents by Bis-aniline-Cross-Linked Pt Nanoparticle/Photosystem I Composites on Electrodes. *J. Phys. Chem. B* **2010**, 114, 45, 14,383–14,388. Copyright (2010) American Chemical Society

DCPIP is popularly utilized in conjunction with ascorbate salts as the sacrificial donor to promote electron transfer for PSI. For instance, Yehezkeli et al. utilized the DCPIP/ascorbic acid pair to reduce the P_700_^+^ redox active center while the electric contact between PSI and anode was maintained by crosslinks of bis-thioaniline (Fig. 4) [65]. The paired system is utilized since DCPIPH_2_, obtained after ascorbic acid oxidation and two-proton-two-electron transfer to DCPIP, is an efficient electron donor for the P_700_^+^ redox active center [43, 66]. Comparatively, ascorbate alone is a poorly efficient electron donor for P_700_^+^, even at high concentrations [67–69]. There is extensive literature on the use of the DCPIP/ascorbate pair as a sacrificial agent or for redox mediation [6, 36, 43, 70–79]. The popularity of DCPIP is partially attributed to PSI enzymes being one of the most frequently used photosynthetic entities in biophotovoltaic systems, with which DCPIP shares bio- and redox potential compatibility. TMPD or Wurster’s Blue follows a similar electron donor–acceptor mechanism to reduce P_700_^+^, though DCPIP is the more efficient donor of the two [64]. Chen et al. investigated the effect of overpotential on photocurrent of solely DCPIP mediated systems of spinach PSI. A − 100 mV overpotential generated a 35-fold current increment, possibly due to the applied potential forming DCPIPH_2_ [17].

Chen et al. also showed that redox mediators that absorb light within the Qy transition band of chlorophylls (650–750 nm) such as DCPIP and MB, compete with light absorbance at P_700_ in PSI, resulting in lowered photobioelectrocatalytic activity. Conversely, MET performance of mediators, which have overlapping absorbance with the Soret band in the blue region of chlorophylls, was relatively unaffected due to the Soret band absorbance not being significantly involved in the initial P_700_ photoexcitation. Therefore, additional considerations have to be made regarding the color of redox dyes as redox mediators for photobioelectrocatalytic systems.

#### Methyl viologen and other bipyridinium salts

**Fig. 5:**
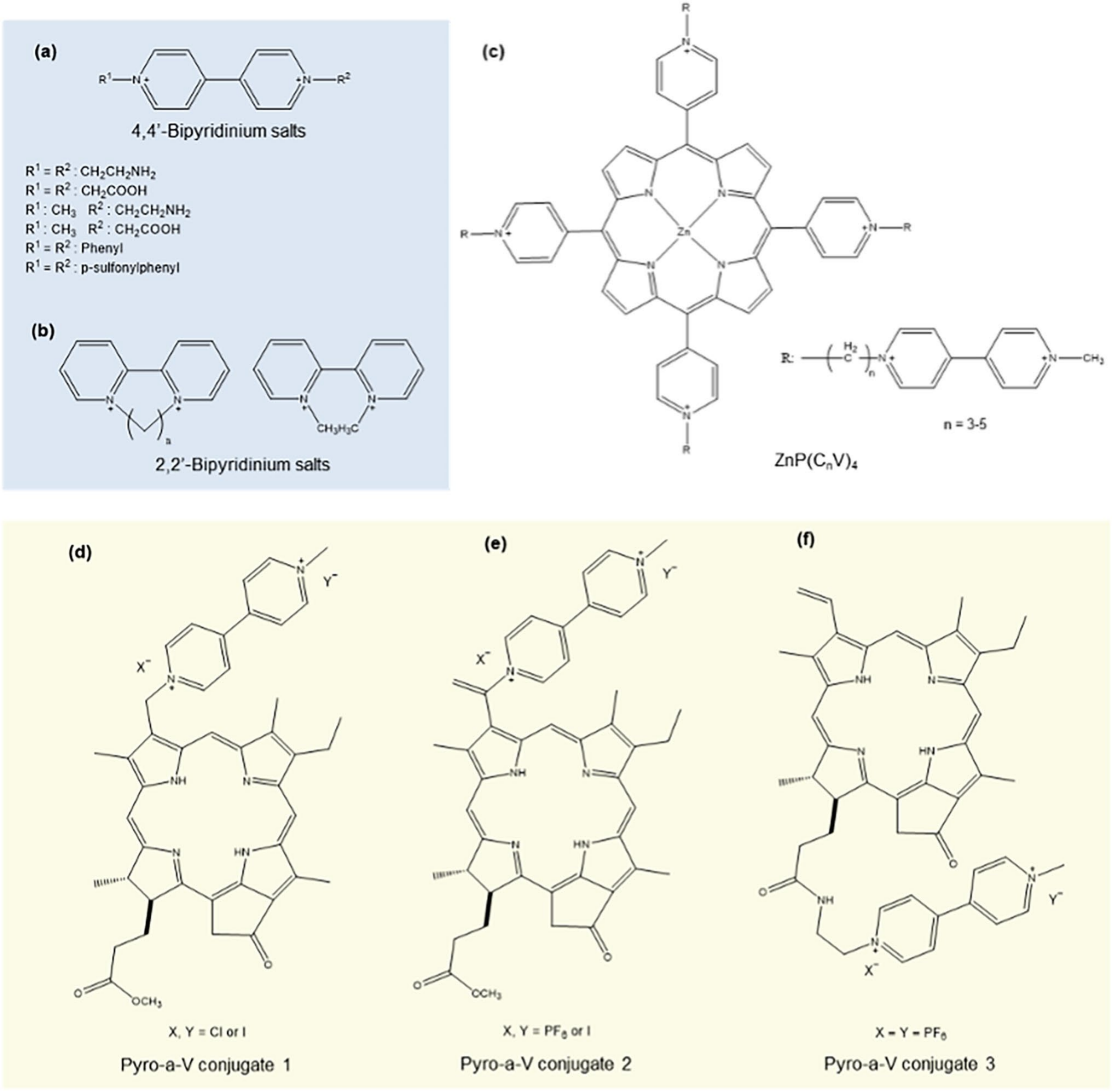
Bipyridinium diffusible redox mediators; **a** 4,4’-bipyridinium salts, **b** 2,2’-bipyridinium salts, **c** viologen-bound cationic porphyrins ZnP(C_n_V)_4_, **d–f** pyropheophorbide-a structures conjugated with viologen

MV has a rich history as a redox mediator in photo- and photobioelectrocatalysis, used typically in conjunction with O_2_ or a secondary sacrificial agent [80–82]. Records of utilizing MV as a redox shuttle for chloroplasts in photobioelectrocatalysis extend as far as the year 1983 [83]. In 2016, Bennett et al. contributed to the mechanistic purview of redox mediation by MV paired with O_2_ during photo-induced electron transfer via PSI of *Thermosynechococcus elongatus * [18]. They proposed the formation of an ephemeral metastable intermediate [MV-oxygen] complex that directly scavenges high-energy electrons from the Fe–S cluster of PSI, producing MV and H_2_O_2_, as opposed to the purported reduction of MV^+^ by O_2_. Bipyridinium salts are also used as redox mediators between porphyrins and enzymes during solar energy-driven CO_2_ reduction to formic acid (Fig. 5a, b) [84–86]. As one of the early-explored redox mediators, the electrochemistry of bipyridinium salts during formic acid production has been thoroughly elucidated [87]. Viologen-bound metal porphyrins are known to serve the dual function of photosensitizer and electron carrier during photo-induced H_2_ production. (Fig. 5c) [87–91]. Photobioelectrocatalytic performance of these synthetic conjugates is impeded by recurrent electron–hole recombination due to poor separation of electron densities and close spatial proximity between porphyrins and bound MV. Ikeyama et al. reported the use of external, freely-diffusing MV with viologen-bound porphyrins to alleviate this complication and facilitate efficient redox mediation (Fig. 5d–f). The Pyro-a-V, constituting conjugated MV and supplemented with diffusible MV, produced 6.7 × 10^–7^ mol of H_2_ upon 120 min of irradiation [39].

**Fig. 6:**
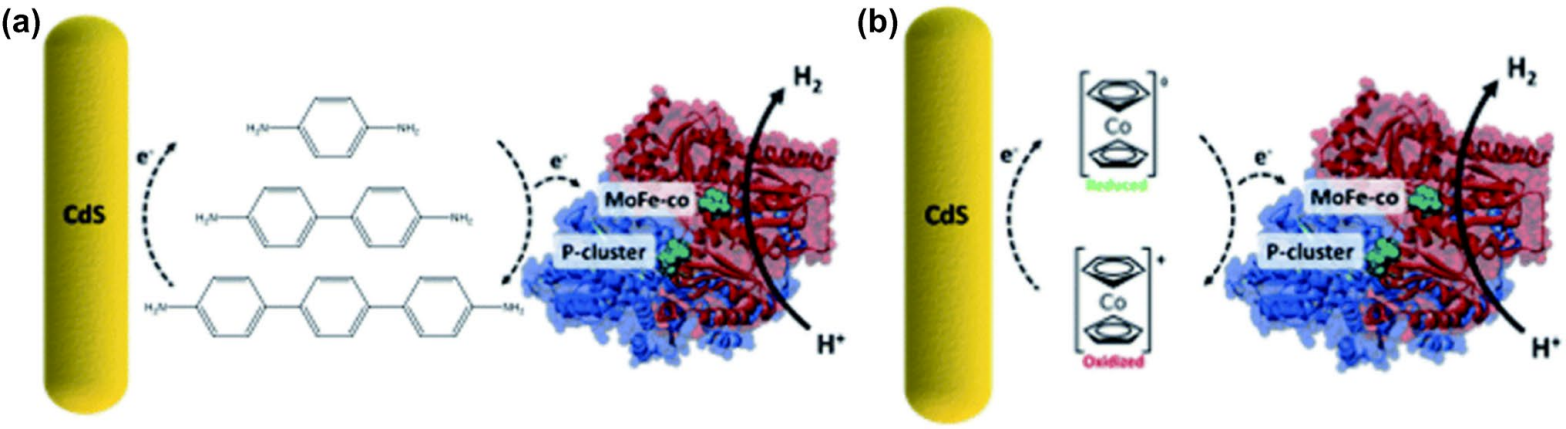
Schematic representation of the electron mediation at the MoFeP enzyme-CdS biotic abiotic interface; **a** oligophenylenes as mediators, **b** cobaltocene as mediator. Adapted with permission from A. Harris, S. Roy, S. Ganguly, A. Parameswar, F. Lucas, A. Holewinski, A. Goodwin and J. Cha. Investigating the use of conducting oligomers and redox molecules in CdS–MoFeP biohybrids. *NanoscaleAdv.,2021*,3,1392. Published by The Royal Society of Chemistry

LHCs of chloroplasts have the ability to function as photocatalysts in vitro [92–94]. Therefore, several biohybrid systems constituting the LHCII trimer or a recombinant LHCII monomer, paired with a sacrificial electron donor, an electron mediator and a catalyst have been reported for photoenergy conversion [95–98]. In 2020, Kondo et al. utilized MV in the dual capacity of redox mediator and mechanistic tool, to respectively compare the photocatalytic performances of the LHCII dimer and the native LHCII trimer during H_2_ production [99]. The dimer allowed superior initial reduction rate of MV and turnover number (respectively 1.95 mM h^−1^ and 143.1) compared to the native trimer (0.52 mM h^−1^ and 59.5) due to better electric contact mediated by MV.

#### Phenazine

Phenazines are small, lipophilic molecules, which have been used for redox mediation during bioelectrocatalysis in *Pseudomonas* and *E. coli*. In 2021, Zhang et al. reported the first use of phenazines as diffusible redox mediators specifically in conjunction with cyanobacteria (*Synechocystis* sp. PCC 6803) in biophotovoltaics [100]. Redox potentials of phenazines generally range high enough to facilitate electron transfer from the cyanobacteria and low enough to cause marginal energy losses in overpotential. Among unsubstituted phenazine, 1-hydroxyphenazine, phenazine-1-carboxylic acid, and pyocyanin (Fig. 3p), only pyocyanin exhibited redox mediation, achieving a 4-fold photocurrent density increment. The poor performance of the other three phenazines was attributed to poor solubility and/or poor electron transfer capacity, and not poor cell permeability as the calculated partition coefficient clog *D* value for all phenazines were high at the thylakoid lumen pH. Spot assays indicated that exogenously added pyocyanin at < 200 μm, and pyocyanin precursor phenazine-1-carboxylic acid at < 500 μm are non-cytotoxic to cyanobacterial growth. The site of mediation by pyocyanin and DCBQ were probed using 3-(3,4-dichlorophenyl)-1,1-dimethylurea and methyl viologen photosynthetic electron transfer chain inhibitors. Mediator longevity of DCBQ and pyocyanin were compared in terms of the photocurrent stability over time. Cyclic voltammetry and UV–vis spectroscopy under light and dark conditions were utilized to compare the molecular stability of pyocyanin and DCBQ. While both mediators undergo different mechanisms that decline the photoactivity of the cell over time, pyocyanin was deemed more chemically stable than DCBQ. Leveraging the extensive genomic and biosynthetic information available for phenazines [101, 102], the study explored the endogenous production of phenazines in *Synechocystis* sp. PCC 6803 using genetic engineering. However, significant concentrations of pyocyanin were not achieved to facilitate redox mediation.

#### *Ferricyanide/ferrocyanide [Fe(CN)*_*6*_^*3−*^*/[Fe(CN)*_*6*_^*4−*^*]*

Inorganic redox mediators bear the advantages of lacking lipophilic moieties that can pose toxicity on biological entities by uncontrollable bilayer permeation, and generally higher diffusion coefficients compared to bulky organic equivalents [17]. Ferricyanide based diffusible and immobilized species are among the most commonly used redox mediators in bioelectrocatalysis [29]. However, the use of ferricyanide in photobioelectrocatalysis is riddled by its toxicity, chemical hazards, and light sensitivity [103, 104].

In 1969, Knaff et al. reported the use of ferricyanide to probe the photoactivity of cytochrome 550, due to its stoichiometric O_2_ production simultaneous to chloroplast photoreduction, and the ability to photo-reduce multiple chloroplast constituents [105]. Tanaka et al. utilized ferricyanide as the redox mediator in the catholyte and *Anabaena variabilis* with HNQ as redox mediator in the anolyte of a fuel cell for the light-influenced reduction of carbohydrates to electricity [46]. Daeneke et al. utilized ferricyanide as the redox shuttle between photooxidized carbazole dye and cathode in a dye-sensitized solar cell (DSSCs) containing an aqueous electrolyte [106]. Compared to the conventional I^−^/I_3_^−^ redox shuttle used in organic nitrile electrolyte-based DSSCs, ferricyanide does not cause undesirable iodate formation and corrosiveness. Despite achieving unparalleled energy conversion efficiencies at the time (4.1 ± 0.1 0.2%), ferricyanide redox shuttle in the DSSC was impeded by higher photoelectron recombination rates compared to I^−^/I_3_^−^. In 2019, Fan et al. extended the redox mediating capacity of ferricyanide into extracting and storing energy from transient singlet oxygen [107]. Type II photosensitization to generate singlet oxygen from O_2_ as a reactive oxygen species (ROS) is used for various organic and drug synthesis, and photodynamic technologies. Singlet oxygen has a lifetime shorter than 4 μs. In the system designed by Fan et al., oxygen was photosensitized by a double strand DNA-SYBR Green I complex, and luminol chemiluminescence was used to analyze the sequestered oxidizing capacity by the redox mediator. Ferricyanide achieved an approximate 30-fold luminol chemiluminescence signal increment, the highest among other transition metal based redox species and I^−^/I_3_^−^. The superiority of ferricyanide performance was attributed to its low redox potential and the ability to induce luminol chemiluminescence.

Wang et al. reported a quintessential biohybrid of self-assembled photocatalysts constituting PSII for efficient water oxidation and inorganic semiconductors (Ru_2_S_3_/CdS and Ru/SrTiO_3_:Rh) for proton reduction, in order to collectively achieve solar-to-H_2_ energy conversion [108]. These biohybrid photosystems were electrically wired via [Fe(CN)_6_^3−^/[Fe(CN)_6_^4−^], producing 2489 mol PSII^−1^ h^−1^ of H_2_ during solar-driven water splitting. In spite of the presence of the ferricyanide redox mediator, the water splitting performance of the Ru_2_S_3_/CdS hybrid was inferior to that of Ru/SrTiO_3_:Rh owing to the hydrophobicity of the former’s surface.

### Complex redox mediator systems

In 2021, the first report of utilizing short, soluble conductive, artificial molecular wires (phenylene based diamines) in order to facilitate electric contact in biohybrids of photoactive nanoparticles (CdS nanorods capped with thioglycolic acid) and enzymes (the hetero-tetrameric molybdenum-iron, MoFe, protein isolated from *A. vinelandii*) was made by Harris et al. (Fig. 6a) [38]. This system generated a 3-fold H_2_ production increment compared to a system without artificial mediation. They further investigated the electron mediation by a series of consistently elongating oligophenylene amines, because the conductance tunneling decay constants of oligothiophenes have been shown to decay less exponentially than across alkanes [109]. While a linear correlation between oligophenylene amine length and catalytic performance was unapparent, the observations were explained in terms of HOMO–LUMO gaps and the reorganization energies of each mediator species [110]. The same CdS-MoFeP biohybrid showed drastically reduced photoactivity upon substituting the oligophenylene amines with profiled organometallic cobaltocene species (Fig. 6b). The poor redox mediation by the diamine- and diacid- cobaltocenes were attributed to their formation of tight binding interactions with the biohybrid, highlighting the significance of the mobility of diffusible redox mediating species. Similarly, there are numerous studies that exploit the efficient redox mediation capabilities and novel approaches based on MV derivatives in order to further photobioelectrocatalysis [25, 29, 111].

**Fig. 7:**
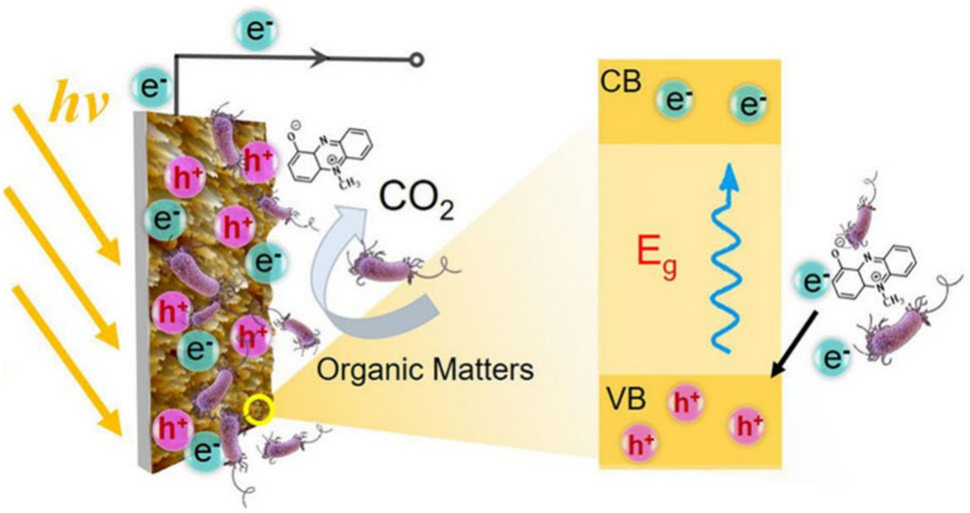
Schematic representation of redox mediation by pyocyanin biosynthesized by *P. aeruginosa*. Adapted with permission from G. Ren, Y. Sun, Y. Ding, A. Lu, Y. Li, C. Wang, and H. Ding, Enhancing extracellular electron transfer between *Pseudomonas aeruginosa* PAO1 and light-driven semiconducting birnessite. *Bioelectrochemistry*, 2018, **123**, 233–240. Copyright (2018) Elsevier

There has been a relatively recent surge in biohybrids of non-photosynthetic biofilms and abiotic photosensitizers in photobioelectrocatalysis in order to generate photocurrent, recycle pollutants, and/or produce energy rich chemicals [112–116]. These biohybrids constitute inorganic semiconductors such as TiO_2_ [117–119], CdS [120–122], Rutile [123], Hematite [112, 113], CuInS_2_ [124], α-Fe_2_O_3_ [114] and organic dyes such as Eosin Y [125] in the photosensitizer capacity, paired to bacteria such as *S. oneidensis, T. denitrificans, E. coli, M. barkeri*. While such systems bear the advantage of tunability (e.g., the ability to synthetically tune the bandgap of a semiconductor to facilitate the maximum solar absorbance [126]), they are nevertheless limited by poor electric wiring to the biofilms, which in turn can be overcome by redox mediation. In 2018, Ren et al. reported the generation of a 279.57 µA cm^−2^ photocurrent density by a biohybrid of *Pseudomonas aeruginosa* on a phyllomanganate birnessite photoanode, where the role of the microbe was redox mediation. The 170% photocurrent density increment as opposed to a system devoid of *P. aeruginosa,* was attributed to biosynthesized pyocyanin-based redox mediation (Fig. 7) [116, 127]. Similarly, several electrochemically-active microbes secrete phenazines to function as diffusible redox mediators [30–32].

In 2018, Xie et al. investigated five biomimetic, porphyrin-based redox mediators for electron mediation in heteromorphic denitrification bacteria [128]. The metallo-porphyrins showed superior denitrification performance compared to non-metallic tetraphenylporphyrin (TPP), in line with the findings of Chen et al., who reported that overall, metal-based diffusible redox mediators undergo faster electron transfer kinetics compared to purely organic species [17]. The denitrification rate in the presence of metallo-porphyrin Hemin was 2-to-3-fold higher and the activation energy was reduced by 87% as opposed to an unmediated system. Zhang et al. reported the use of [Cp*Rh(bpy)H_2_O]^2+^ (Cp* = pentamethylcyclopentadienyl, bpy = 2,2′-bipyridyl) as redox mediator at the enzyme-CdS/PTi microcapsule interface during CO_2_ conversion. The resulting system recorded a NADH regeneration rate of 4226 ± 121 μmol g^−1^ h^−1^ [74]. Analogously, many other reports of azo-, ferricyanide-, and methylene blue- mediated photobioelectrocatalysis are available [54, 56, 129, 130].

Incorporating diffusible redox mediators to photobioelectrocatalytic cells is a straightforward means of implementing exogeneous E-MET, in order to obtain relatively significant photocurrents (vide supra). Certain reports attribute the use of mixed-diffusible mediator systems as a means of emulating the natural photosynthetic operations where chains of redox centers participate congruously [17]. However, such mixed-mediator system design instigate energy loss and higher overpotentials. Additionally, the applicability of many available redox mediators is limited by their inherent toxicity or unstable nature [71, 131], and slow kinetics [132]. Certain mediators can cause cell death by intercepting the electron transfer of important metabolic events [58, 133]. Diffusible redox species cannot provide an immobilizing matrix for biotic components at the biotic-abiotic interface, which necessitates extrinsic agents. Such immobilizing agents include nanoparticles [58], CNTs [134], crosslinkers (e.g.,EGDGE [135], cytochromes [136]), SAMs with terminal metal-nitrilotriacetic acid complexes [52, 137], *N*-hydroxysuccinimide (NHS), terephthaldehyde and other –OH, –NH_2_, –CH_3_ bearing groups for covalent or electrostatic interactions [70, 81, 114, 138].

## Exogenous E-MET via redox polymers

**Fig. 8:**
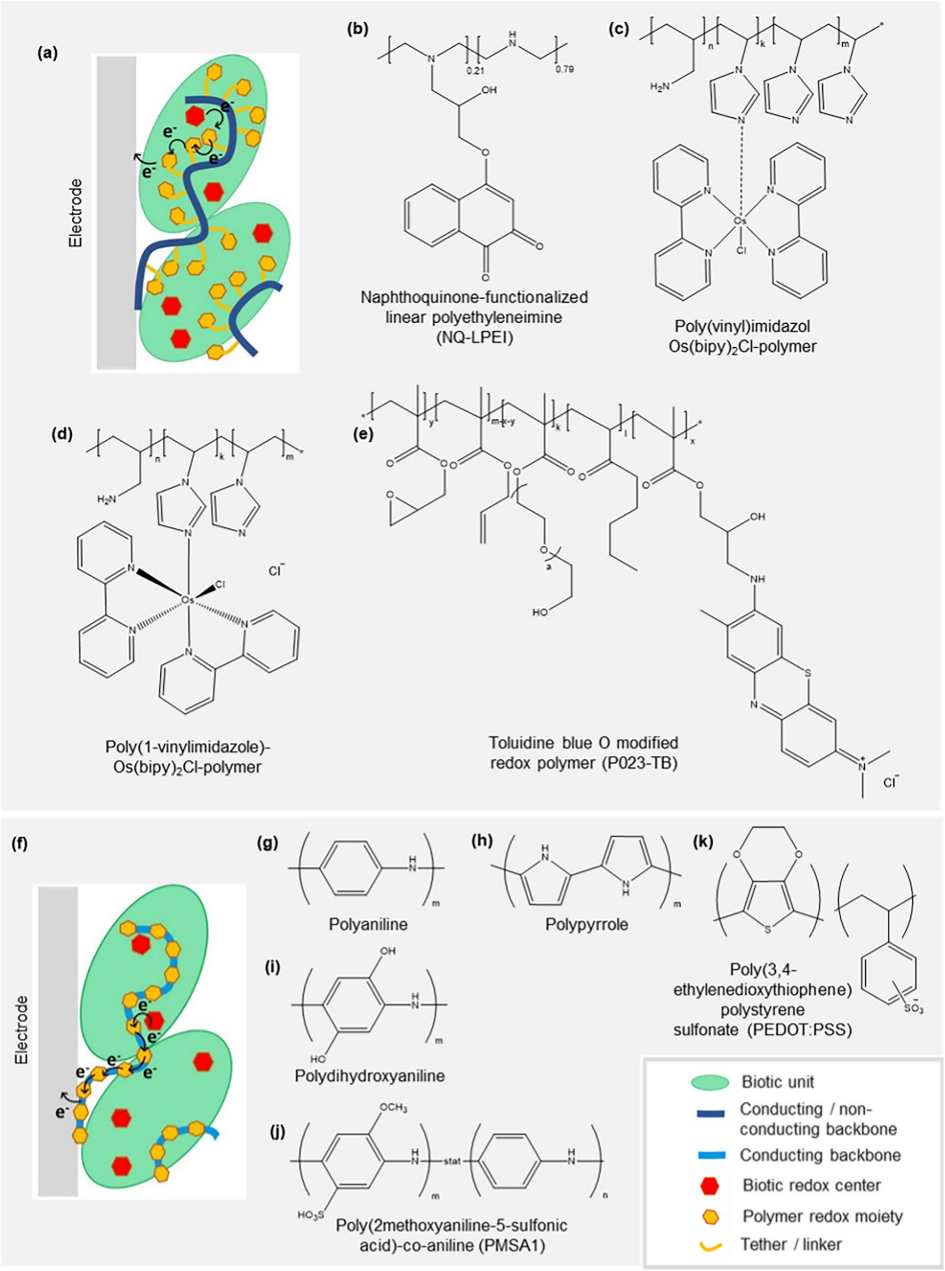
Redox polymers by a structural classification; **a** schematic representation of generalized electron transfer in a branched redox polymer, **b–e** examples of branched redox polymers, **f** schematic representation of generalized electron transfer in an unbranched redox polymer, **g–j** examples of unbranched redox polymers

The diverse complexity of biotic components, which are isolated or engineered for specific applications in photobioelectrocatalytic biohybrids, necessitates sophisticated complementary redox mediating species for efficient E-MET. Compared to the relatively simple composition of diffusible redox mediators, redox polymers provide a more expansive chemical architecture to modulate as per requirement. Specifically, individual components of redox polymers; the redox pendant, polymer backbone, and the tether, can be chemically tuned in order to thermodynamically and kinetically optimize electron transfer (Fig. 8a, f) [139]. The advancement of redox polymers in photobioelectrocatalysis will be deconstructed by chemical structure and components in this section. Readers are directed elsewhere for redox polymers functioning solely as photocatalysts [140].

The formulation of redox polymers restricts the mobility of redox pendants, and the consequent polymer structures can be broadly classified into branched and unbranched redox polymers (Fig. 8). The redox pendants are resonant with diffusible redox mediators and can either be fully organic molecules or transition metal-based organometallic complexes. For instance, methylene blue diffusible mediators have been electropolymerized on electrode surfaces [141]. Branched redox polymers consist of tethers or linker arms that tie redox pendants to a conducting or non-conducting polymer backbone, rendering the said pendant a capacity for “bounded diffusion” [75]. Unbranched redox polymers have redox pendants embedded into their conducting polymer backbones, “immobilizing” the redox couple in the electron density of the backbone. Both the “bounded diffusion” and the “immobility” of redox pendants in each polymer type prevent their leaching into the periphery of the cell, which allows redox polymer application in the body, in flow cells, and in miniature, compartmentless biofuel cells [75]. Redox polymers deposited on the electrode surface provide consistent physical contact compared to the diffusible redox mediators at the biotic-abiotic interfaces. Additionally, modulating the functionalization of the redox polymer to complement the surface hydrophobicity/hydrophilicity and the surface charge of the photosynthetic entity can enforce electrostatic, covalent, affinity interactions or entrap biotic units in an immobilization matrix [36]. Redox polymer deposition methods include adsorption, blending with activated carbon, layer-by-layer assembly, electropolymerization, or self-assembled monolayers by covalent linkages [142, 143].

Hydrogel formation by the electrically conducting polymer matrices creates a hydrophilic environment that allows wettability by aqueous electrolytes. The hydrophilicity-hydrophobicity balance in the ensuing system enforces high catalytic loading and rapid electron and mass transport of water-soluble species [144]. Redox polymer designs can be made receptive to auxiliary signals such as pH, temperature, ionic strength, and chemical species within the vicinity, by incorporating structural modifications. For instance, Ruff et al. utilized viologen-modified polymers in conjunction with [NiFeSe] hydrogenase from *Desullfovibrio vulgaris* for the oxidation of H_2_ [145]. The viologen moiety functions as an O_2_ scavenger, protecting [NiFeSe] hydrogenase and reactivates the inhibited enzyme, resulting in an overall current density of 1.7 mA cm^−2^.

### Branched redox polymers

A large fraction of redox polymers applied in photobioelectrocatalysis falls in the “branched” category. In branched redox polymers with a nonconducting backbone, the redox pendant permeates through membranes or into deeply-welled redox active centers, using the “bounded diffusion” imparted by the tether of the polymer, in order to extract electrons. Subsequently, Marcus-type collision-based electron tunneling propagates these electrons across the polymer. In branched redox polymers with conducting backbones, the redox pendants and backbone synergistically facilitate electron transfer, which depends on the redox potentials of both the pendant and backbone being similar and the tether length being short enough to maintain considerable electron delocalization [132]. The significance of redox pendant potential and steric effects during the electron extraction from catalytic sites is denoted for example, by phenothiazine redox pendants being known for electron mediation from the FAD-dependent dehydrogenase domain of cellobiose dehydrogenase [146, 147]. Guschin et al. developed a new library of the first generation Os-based redox polymers by modifying ligand substitutions. Corresponding redox potential changes and electron transfer rates during electron wiring at biotic-abiotic interfaces were investigated [148, 149]. As previously mentioned, redox polymers need to strike a hydrophobicity-hydrophilicity balance in order to maintain good contact with the generally aqueous electrolytes and predominantly hydrophobic biotic components. Zhao et al. tuned the hydrophobicity of Os-based redox polymers to enforce hydrophobic interactions with PSI by mutual attractive association [150]. They modulated the hydrophobicity by adding various hydrophobic moieties to substitution groups around Osmium and the polymer backbone, successfully developing a general trend. A pH-triggered switchable collapse of the redox polymer to better encapsulate PSI, based on the Lewis basicity of pyridines [150], and imidazole [144] in Os-based redox polymer backbones was also developed. Collapsed Os-redox polymers with the imidazole-based backbone reported a 322 ± 19 µA cm^−2^ photocurrent density and an electron transfer rate of 335 ± 20 e s^−1^ PSI^−1^ [144]. Karlsson et al. investigated the effect of conjugation and atomic length of tethers between a hydroquinone redox pendant and a polypyrrole backbone, in terms of polymer packing and consequent electron transfer capabilities [132]. Mao et al. developed an Os-based redox polymer with 13-atoms long tethers that had an apparent electron diffusion coefficient (*D*_app_) of 5.8 ± 0.5 × 10^6^ cm^2^ s^−1^ owing to the elevated mobility of the tether and better hydrogel formation [132].

Milton et al. synthesized a series of naphthoquinone-based redox polymers on linear polyethyleneimine backbones (NQ-LPEI) in order to electrically wire a number of different biotic-abiotic interfaces (Fig. 8b) [48, 151]. The nature of the tether and its substitution position on the naphthoquinone was shown to impact the catalytic performance. NQ-LPEI was used to wire chloroplasts, which are one of the most electrically insulating membranous photosynthetic units due to high order compartmentalization, onto electrodes in biophotovoltaic cells. The resulting biohybrid generated a maximum of 4.7 ± 0.7 µA cm^−2^ photocurrent density that elevated to 29 ± 6 µA cm^−2^ upon being simultaneously supplemented by the diffusible redox mediator DCBQ. Grattieri et al. electrically wired *Rhodobacter capsulatus* to Toray carbon electrodes via NQ-LPEI, resulting in a biohybrid that operated at + 0.317 V vs SHE as opposed to leading Os-based redox polymers, which operated at + 0.547 V vs SHE [152]. The significant decrease of overpotential was attributed to the comparability of redox potentials of the two redox polymers and the redox centers in the photosynthetic organisms.

Badura et al. reported Os(bpy)_2_Cl-modified poly(vinyl)imidazole redox polymer hydrogels to electrically contact less-membranous PSII [153] and PSI [73] of *Thermosynechococcus elongatus,* respectively, to gold electrodes (Fig. 8c). PSII-Au system yielded a 10-fold current increment at 45 µA cm^−2^ and improved lifetimes owing to superior stability. The PSI-Au system yielded a 29 µA cm^−2^ photocurrent density upon being further supplemented by MV, resulting in a photon to carrier efficiency of 3.1%. In 2016, Sokol et al. compared the individual performance of an Os-based, and a phenothiazine-based redox polymer for the electric wiring and catalytic loading of PSII onto OI-ITO (Fig. 8d, e) [154]. The Os-based redox polymer evinced the superior performance with a photocurrent approaching 410 µA cm^−2^ at 0.5 V vs. SHE. Although the phenothiazine-based redox polymer had a more comparable redox potential to *Q*_A_ and *Q*_B_, it showed lower photocurrents due to lower adsorption stability on the IO-ITO electrode, lower driving force for electron transfer owing to a more negative redox potential, and slower proton-coupled-electron transfer in the absence of organometallic redox centers. On the other hand, biohybrids with green algae *Paulschulzia pseudovolvox*-graphite interfaces have reported Os-mediated photocurrent densities of 0.44 µA cm^−2^, which is significantly lower compared to biohybrids of *Rhodobacter capsulatus* and *Leptolyngbya* sp., with Os-based redox polymers as mediators [155]. This performance has been elevated to 6.97 µA cm^−2^ by supplementing the redox polymer with benzoquinone. Zhao et al. utilized a PSI-Pt biohybrid electrically wired by poly(vinyl)imidazole Os(bispyridine)_2_Cl that facilitated the hydrogen evolution reaction at lowered overpotentials due to the concomitant photocurrent generation [139, 150].

**Fig. 9:**
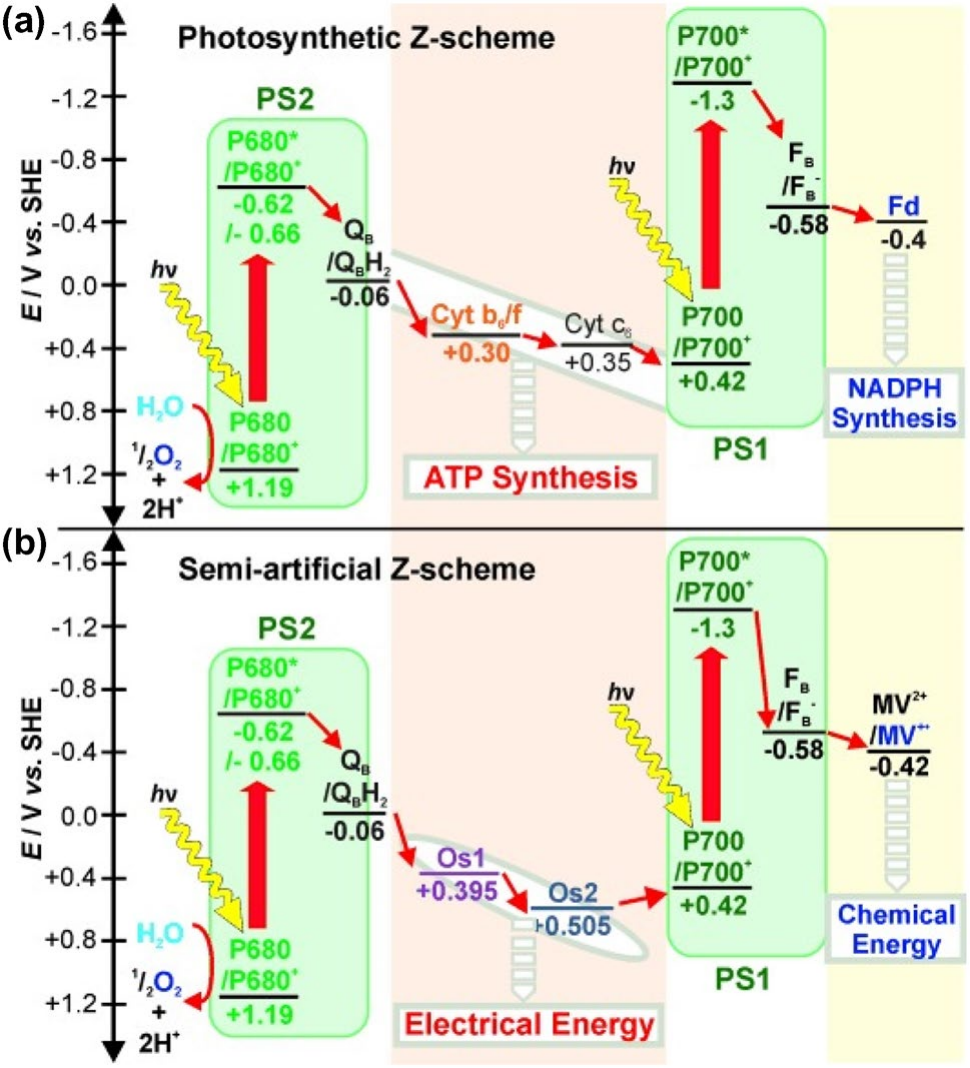
Schematic representation of electron transfer in the Z-scheme; **a** during natural photosynthesis, **b** semi-artificial Z-scheme, where PSII-PSI interface is redox mediated by Os-redox polymers. Adapted with permission from T. Kothe, N. Plumeré, A. Badura, M. Nowaczyk, D. Guschin, M. Rçgner, and W. Schuhmann, Combination of a Photosystem 1‐Based Photocathode and a Photosystem 2‐Based Photoanode to a Z‐Scheme Mimic for Biophotovoltaic Applications. *Angew. Chem., Int. Ed.*, 2013, **52**, 14,233–14,236. Copyright (2013) Wiley

Kothe et al. designed a semiartificial *Z*-scheme utilizing an imidazole coordinated bispyridyl osmium complex-based redox hydrogel and a pyrdine coordinated bispyridyl osmium complex-based redox hydrogel to electrically wire PSII and PSI (Fig. 9) [156]. The power output of the resulting biophotovoltaic system was a function of the redox potentials of the osmium redox hydrogels. Hartmann et al. recreated the semiartificial *Z*-scheme using two other redox polymers in an effort to reduce the potential difference between them [149]. An Os-based redox polymer with a redox potential that closely matched the donor site of PSI, and generated high current densities, was used to electrically wire PSI. Eighteen different redox polymers based on four individual redox dye pendants; toluidine blue (TB), nile blue (NB), azure blue (AzB), neutral red (NR), and poly(ethylene glycol) methacrylate-containing backbones were screened to electrically wire PSII. TB, NB, AzB, NR are phenothiazines with redox potentials that match Q_B_ of PSII. Achievable photocurrent densities of PSII-phenothiazine-based redox polymers were directly related to the redox pendant potential and the hydrophilicity of the backbones. The TB-containing redox polymer (Fig. 8e) wired to PSII in tandem with the Os-based redox polymer wiring PSI, generated a 125-fold performance enhancement (an energy conversion efficiency (*η*) of 0.0045%, maximum power output 1.91 ± 0.56 mW cm^−2^) compared to previously reported semiartificial *Z*-scheme systems. Similar systems have been further investigated since, for better photobioelectrocatalytic performance [154].

**Fig. 10:**
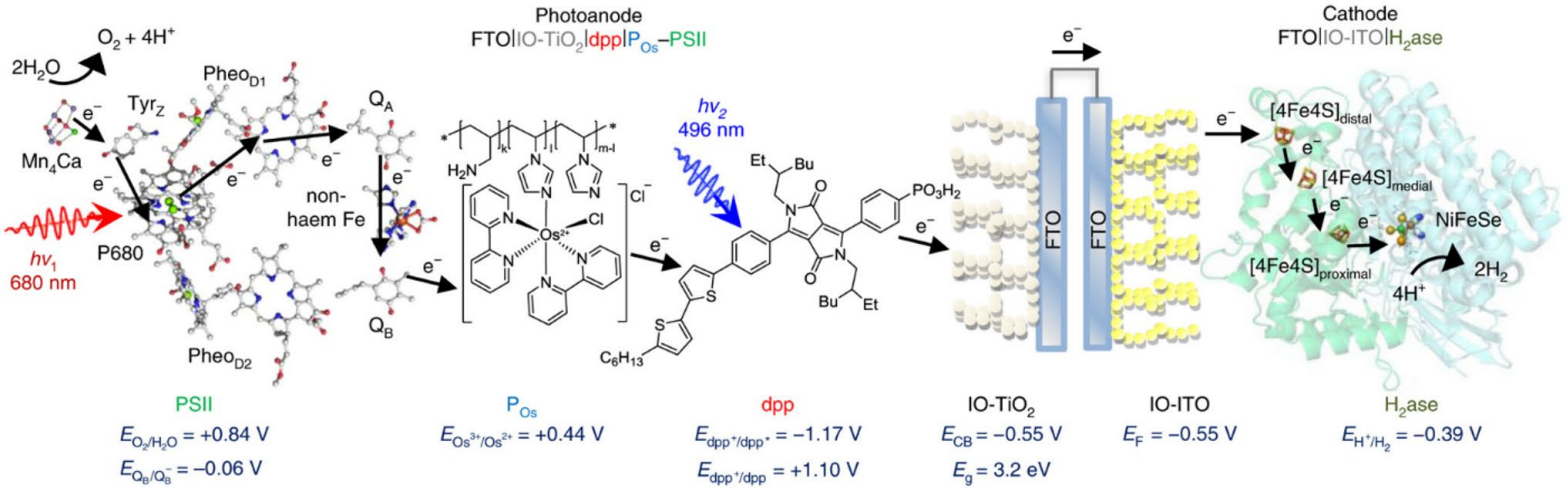
Schematic representation of electron transfer at the PSII-diketopyrrolopyrrole dye interface, where both biological entities harvest solar energy, en route to the hydrogenase enzyme. Adapted with permission from K.P. Sokol, W. E. Robinson, J. Warnan, N. Kornienko, M. M. Nowaczyk, A. Ruff, J. Z. Zhang and E. Reisner. Bias-free photoelectrochemical water splitting with photosystem II on a dye-sensitized photoanode wired to hydrogenase. *Nat. Energy,* 2018, **3**, 944–951. Copyright (2018) Nature

In 2018, Sokol et al. reported a multicomponent semiartificial photosynthetic system for bias-free solar-driven water splitting via a [FeFe]-hydrogenase (Fig. 10) [157]. The photoanode of the system constituted PSII, which absorbs red and blue light, and diketopyrrolopyrrole dye sensitized TiO_2,_ which absorbs green light. Electrical contact between PSII and diketopyrrolopyrrole dye-sensitized TiO_2_ was maintained by an Os-based redox polymer. The slightly unconventional redox mediating role fulfilled by the Os-based redox polymer in this tandem system highlights the potential of redox mediation to overcome one of the biggest challenges in photobioelectrocatalysis and simultaneously facilitating panchromatic solar absorbance.

**Fig. 11:**
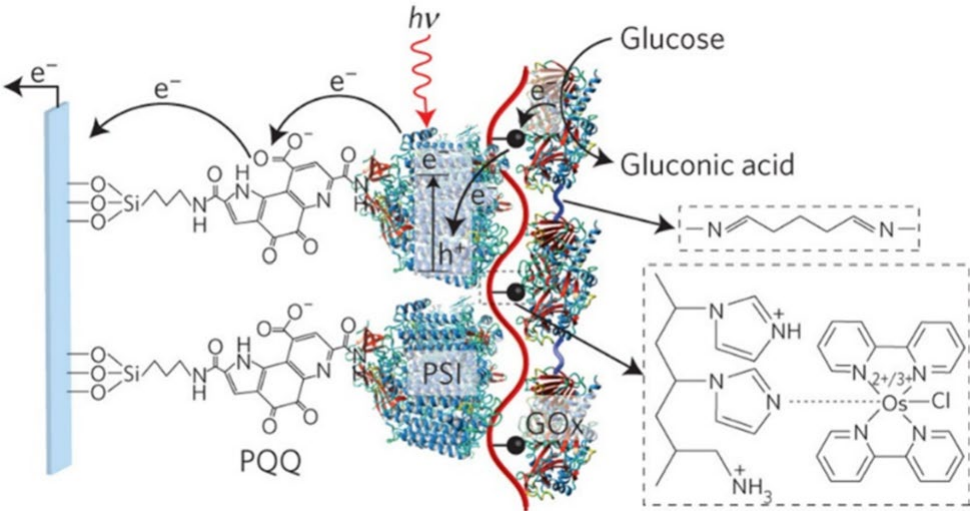
Schematic representation of the photobioelectrochemical enzymatic electrode incorporating a glucose oxidase-PSI interface electrically wired by an Os-based redox polymer, and the PSI-ITO electrode interface electrically wired by pyrroloquinolinequinone. Adapted with permission from A. Efratil, C. Lu, D. Michaeli, R. Nechushtai, S. Alsaoub, W. Schumann and I. Willner. Assembly of photo-bioelectrochemical cells using photosystem I-functionalized electrodes. *Nat. Energy,* 2016, **1**, 15,021. Copyright (2016) Springer Nature

On the other hand, Riedel et al. developed a FAD-dependent glucose dehydrogenase-PbS quantum dot photosensitized IO–TiO_2_ biohybrid in an effort to harvest solar energy into sensing and electrical energy [158]. The enzyme-PbS interface was electrically wired by an Os-based redox polymer, which resulted in a maximum photocurrent density of − 207 µA cm^−2^, and the lowering of the working potential by over 500 mV compared to light-insensitive electrodes. In 2016, Efrati et al. developed a photobioelectrochemical signal cascade by photonically-wiring glucose oxidase enzyme to PSI via the electrostatic interactions of poly(vinyl)imidazole Os(bipyridine)_2_Cl polyallylamine copolymer [77]. PSI, in turn, was electrically wired onto ITO electrode via the covalent linkages of a pyrroloquinoline quinone monolayer (Fig. 11). Interestingly, the electric mediation, in this case, is instigated at a biotic-biotic interface. In a parallel sense, Ciorni et al. used redox protein cytochrome C to improve the electric contact between PSI and human sulfite oxidase [159].

### Unbranched redox polymers

Contrariwise to branched redox polymers, the “unbranched” redox polymer category currently is considerably smaller. In unbranched redox polymers the redox moiety is typically embedded in a conductive backbone. The resultant extended electron delocalization reduces the energy barrier for facile electron transfer and facilitates intra- and interchain electron hopping mechanism through a series of discrete redox centers across the polymer [160]. Unbranched redox polymers form hydrogels by interchain stacking and consequent crosslinking, as opposed to branched redox polymers. The lack of “bounded diffusion” of the redox pendant in unbranched polymers is possibly compensated by, either the collective displacement of the redox polymer strands to make direct contact with the photoactive reaction centers due to low steric hindrance or the polymers’ ability to immobilize photoactive reaction centers in close proximity and appropriate orientation to facilitate fast electron transfer, or contribution by endogenous redox shuttles of photosynthetic entities to further electrically wire themselves to polymers. While there is no general consensus on the exact electron extraction mechanism at play with unbranched redox polymers, their high electron conduction ability favors redox mediation.

Zou et al. reported that cathodes coated with a mixture of carbon paint and either polymer polyaniline (Fig. 8g) emeraldine salt or undoped polypyrrole (Fig. 8h) facilitated substantially superior biofilm growth of *Synechocystis PCC-6803* and interfacial electron transfer compared to cathodes without polymer infusions [45]. During 20-days of operation under light, the presence of polymers reduced the drop of potential and the apparent internal resistance. Power density outputs of the polyaniline and polypyrrole systems were respectively 0.95 mW m^−2^ and 1.3 mW m^−2^, which are 171% and 271% increments compared to a bare cathode. HNQ was added to further supplement this polyaniline performance, yielding a 195% increase in the power output. This study also showed that planktonic MFCs require electron shuttles more than biofilm counterparts. The presence of polyaniline and polypyrrole were postulated to either improve the intrinsic conductivity of the biohybrid, or render their polymeric chains to actively extract electrons across membranes from *Synechocystis PCC-6803*. Similarly, Rosenbaum et al. utilized poly(2,3,5,6-tetrafluoroaniline) and poly(2-fluoroaniline) for the electrochemical communication at the green algae *Chlamydomonas reinhardtii*-electrode interface [161].

Weliwatte et al. reported a photocurrent density increment of 2.4-fold in a biophotovoltaic cell of spinach chloroplasts by the use of hybrid conducting redox polymer polydihydroxy aniline (PDHA) (Fig. 8i) [162]. The PDHA-chloroplast combination was deposited in an alternate ‘layered’ method to counterbalance the competition for panchromatic light absorbance by the black colored PDHA, resulting in a 4.2-fold photocurrent density increment. Supplementing PDHA with diffusible mediator DCBQ resulted in the highest photocurrent density reported with intact chloroplasts in biophotovoltaics (− 48 ± 3 μA cm^−2^).

**Fig. 12:**
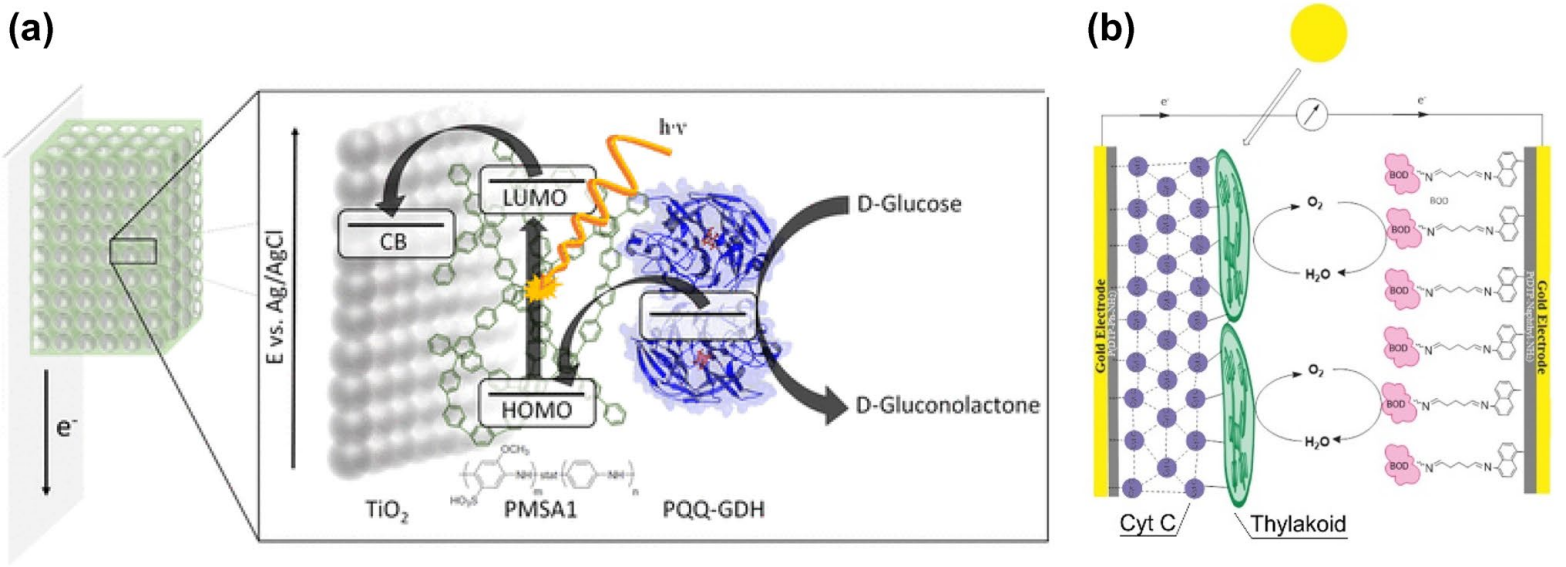
Schematics of electron transfer; **a** at the PQQ glucose dehydrogenase-TiO_2_ interface redox mediated by polymer PMSA1 represented in terms of energy levels. Adapted with permission from M. Riedel, F. Lisdat, Integration of Enzymes in Polyaniline-Sensitized 3D Inverse Opal TiO_2_ Architectures for Light-Driven Biocatalysis and Light-to-Current Conversion, ACS *Appl. Mater. Interfaces*, 2018, **10**, 267–277). Copyright (2018) American Chemical Society. **b** in a photobioelectrocatalytic fuel cell where the thylakoid-Au electrode interface and the bilirubin oxidase-Au electrode interface are respectively redox mediated by conductive poly 4-(4H-dithieno [3,2-b:2′,3′-d]pyrol-4-yl) aniline crosslinked to cytochrome C and poly[5-(4H-dithieno [3,2-b:2′,3′-d]pyrol-4-yl) naphtalene-1-amine]. Adapted with permission from E. Cevika, B. Carbas, M. Senel, H. Yildiz, Construction of conducting polymer/cytochrome C/thylakoid membrane-based photo-bioelectrochemical fuel cells generating high photocurrent via photosynthesis, *Biosens. Bioelectron.*, 2018, **113**, 25–31. Copyright (2018) Elsevier

With the aim to target both electrical wiring at interfaces and panchromatic light absorbance, in 2018 Riedel et al. reported the use of poly(2-methoxyaniline-5-sulfonic acid)-co-aniline (PMSA1) (Fig. 8j) for the first time in the dual role of photosensitizer to TiO_2_ and redox mediating polymer at the PQQ glucose dehydrogenase-TiO_2_ interface (Fig. 12a) [163]. The system achieved a maximum photocurrent of 44.7 ± 6.5 μA cm^−2^ using glucose as fuel, and elevated power output owing to the biohybrid system operating at a lower potential than the autonomous PQQ glucose dehydrogenase. This example consolidates the scope afforded by multifunctional redox polymer designs to systematically overcome the existing limitations of the technology.

In 2018, Cevik et al. developed a photobioelectrocatalytic fuel cell with a compounded photoanode and cathode for photocurrent generation [164]. The photoanode contained thylakoid membranes covalently attached to cytochrome C via organic linkers, which in turn made contact with the electrode surface via covalent interactions with an electropolymerized film of conductive poly 4-(4H-dithieno [3,2-b:2′,3′-d]pyrol-4-yl) aniline (Fig. 12b). Cytochrome C crosslinked to the polymeric oligoaniline, bridged the thylakoid-gold electrode biotic-abiotic interface collaboratively. In addition, the hybridization between conducting redox polymer and natural protein cytochrome C reportedly increased the thylakoid loading onto the gold electrode. The cathode constituted bilirubin oxidase with specific covalent attachments to a conductive film of poly[5-(4H-dithieno [3,2-b:2′,3′-d]pyrol-4-yl) naphtalene-1-amine] via a glutaraldehyde linkage. The presence of the conducting polymers elevated the electron transfer efficiency of the biohybrid by 6-fold. This tandem photobioelectrocatalytic cell architecture further extends the potential roles of redox polymers in photobioelectrocatalysis by facilitating preferential orientations between biotic and biotic units for fast electron transfer, in addition to electrical wiring.

A fairly recent functional extension of redox polymers includes microscale biosolar cells (micro-BSCs). Micro-BSCs hold much promise as prospective self-sustaining, simple, portable, disposable and biocompatible point-of-care devices [165, 166]. Despite intensive research on this pursuit, the first three generations of micro-BSCs have been impeded by internal resistance in the MΩ-range, leading to very slow electron transfer, very low power densities (5.72 pW cm^−2^ [167], 40 pW cm^−2^ [168], 6.05 µW cm^−2^ and 2.7 µW cm^−2^ [169]) and short lifetimes of a few hours to days. Liu et al. developed a micro-BSC using *Synechocystis* sp. PCC 6803 interfaced by poly(3,4-ethylene dioxythiophene): polystyrene sulfonate (PEDOT: PSS), which evinced high electrical conductivity, biocompatibility, and surface affinity for microbial growth (Fig. 8k) [170]. The resulting system showcased a maximum power density of 43.8 µW cm^−2^ and functioned for 20 days, both of which were predominantly attributed to the PEDOT: PSS layer, representing a remarkable advancement for photobioelectrocatalysis in biosensing.

### Alternative redox polymer classification

In bioelectrocatalysis, an alternative classification proposed by Kaneko et al. for redox mediating polymers exists, based on their membrane permeability and diffusivity [171]. Therein, Type I polymeric redox mediators are fully immobilized on the electrode surface, whereas Type II analogs possess a certain degree of cell membrane permeability, which allows the polymer to diffuse across membranes in and out of a cell. Osmium complex-based redox polymers (Fig. 8c, d) fall into Type I. Amphiphilic redox polymers that can form polymer aggregates, micelles, polymersomes, or nanogels in order to permeate through the lipophilic phospholipid bilayers in cells, generally fall into Type II.

Ambiguity of this classification stems from the deficits in mechanistic understanding of electron extraction and transfer by redox polymers during photobioelectrocatalysis. For instance, while quinone-based polymers like NQ-LPEI are considered Type I as they are deposited on electrodes, their peripherally-attached redox pendants are believed to permeate across membranes due to “bounded diffusion” [151, 172]. Intuitively, the strength of interactions between polymer and electrode would determine the degree of freedom for movement of the latter, rendering it immobile, “diffusive” or both.

### Quantification of electron transfer via redox polymers

While the redox potentials of the mediating species govern the thermodynamic feasibility of electron transfer at the biotic-abiotic interface, the apparent electron diffusion coefficient (*D*_app_) is a kinetic parameter that quantifies the rate of electron transfer through the redox polymer (vide supra). Ipso facto, *D*_app_ also correlates to the ability of the redox polymer to electrically wire the biotic-abiotic interface. Holistically, *D*_app_ is a function of the chemical structure of the redox polymer, polymer crystallinity, electrolyte nature, temperature, and, pH among other factors. The majority of redox polymers employed in redox mediation in photobioelectrocatalysis are either fully amorphous or amorphous with infrequent interconnected crystal domains. Interchain coupling in redox polymers can induce crystallinity in the range approaching metallic- or semi metallic-behavior [173]. According to studies so far, for organic redox pendant-bearing branched polymers, *D*_app_ ranges in the low orders of 10^–8^–10^–12^ cm^2^ s^−1^, whereas transition metal complex bearing redox polymers range in the orders of 10^–6^–10^–9^ cm^2^ s^−1^ [75, 144, 174, 175].

Various research groups, including Heller [75, 176, 177], Savéant [178–181], Murray [182–185], Laviron [186], Dahms [187], and Ruff [188, 189] provide a strong seminal foundation on elucidating the *D*_app_ of different redox species by probing either transient currents or steady-state currents. Transient currents obtained via potential step methods across the biotic-abiotic interface are analyzed *approximating* Randles–Sevcik, and Cottrell conditions. Steady-state currents are obtained via either sandwich, rotating ring-disk, or interdigitated array (IDA) electrodes. IDA electrodes have overlapping diffusion spheres between electrodes, rendering the diffusion of macroscopic mobile counterions obsolete in the scheme. However, the electron transfer mechanisms in redox polymer hydrogels and the biotic-abiotic interface tend to deviate from the postulated conditions around which the above methods have been developed (e.g., quasi-reversible, proton-coupled electron transfer in redox polymers, non-planar electrodes, uneven polymer thickness, intricate and subordinate interactions with biotic and abiotic components) [190]. Therefore, incisive application of a host of these methods and consequently, judicious data analysis to determine *D*_app_, are required. For example, Murray and co-workers reported the use of time-of-flight electrochemical measurements on microdevices for molten redox polymer hybrids and corroborated the results with cyclic voltammetry and chronoamperometry [185]. In addition, electrochemical impedance spectroscopy (EIS) can be used to quantify the ohmic loss or overpotential at the biotic-abiotic interfaces, which quantifies the resistance during electron transfer. Somewhat similar to the previous methods, EIS is riddled by the complexity of accounting for all the components of the biotic unit-redox polymer-abiotic unit during data interpretation.

## Challenges in redox mediation and future directions

As discussed in the previous sections, while consistent progress has been made with redox mediation in photobioelectrocatalysis, ubiquitous challenges remain to-date. From a thermodynamic viewpoint, inserting single or multiple redox mediators at the biotic-abiotic interface entails an energy penalty, irrespective of the improvement on the electric contact. Secondly, prospective redox polymer designs are restricted by the need to avert bio- and chemo-incompatibilities with corresponding biotic components. This challenge is exacerbated by the fact that while the complexity of the biotic and abiotic components is being elevated, existing mechanistic understanding of biotic components and especially photoexoelectrogenesis remain incomplete, limited by their intricacy and available methods of analysis. For example, while methylene blue has been used to mediate PSI, potential methylene blue-induced metabolic effects have not yet been explored within the context of photobioelectrocatalysis with different biotic components, to the best of authors’ knowledge [17, 44]. The incomplete understanding of the electron transfer mechanisms also hinders the redox mediator designing and tailoring process. Comprehensive analysis of data and trends is complicated by the multivariant nature of the interactions in increasingly sophisticated biohybrids. Additionally, deleterious effects of exogeneous redox mediators have been reported in terms of generating harmful ROS, intercepting untargeted reactions or mechanisms in biotic components due to poor selectivity, and/or creating adducts of exogenous redox species-membrane components (vide supra) [40, 191–194]. Due to the compounding effect of these factors, the stability and longevity of redox mediated biohybrids in photobioelectrochemical cells remain low. On the other hand, while synthetic chemistry has advanced to accommodate complex redox mediator designs and high scope of structural tunability, batch-to-batch variability of redox polymer synthesis is an existing disadvantage. There is a need for optimized synthetic and deposition techniques for high densities of redox active sites in polymers on electrode surfaces for high catalytic performance [183].

### De novo systematic generation of “ideal” redox mediators

Despite of four decades worth of research, there is a discernible lack of redox-mediated, large commercial scale photobioelectrocatalytic systems. On the other hand, new redox mediator designs for photobioelectrocatalysis are pressed to make additional accommodations for their expanding roles in photobioelectrocatalysis. For example, in addition to efficient electrical wiring, new redox mediators must provision for stability, solar absorption, and explore new chemical conversion possibilities for specific isolated biotic components. Therefore, it is important to streamline the existing knowledge on redox mediators for the rational design of generalized “ideal” counterparts.

In essence, one of the key factors that govern a redox mediator’s ability for reversible electron transfer is its redox potential [195]. Redox potential determines the thermodynamic feasibility of electron extraction from a biotic or abiotic source, unidirectional electron transfer in the desired path, and energy loss during electron transfer at interfaces. In the case of branched redox polymers with conducting backbones and unbranched hybrid conducting-redox polymers, the redox potential of their redox moieties also influences the electron conduction mechanism across the respective polymers, which also necessitates matching redox potentials of redox pendant and backbone in the former. The dependence of formal redox potential on auxiliary factors such as electrolyte, pH, temperature, etc. highlights the significance of judicious pairing of redox mediators, not only with the right biotic-abiotic interface, but also the cell composition in order to avoid thermodynamic overpotential losses.

More photo-harvested energy can be lost during electron transfer due to kinetic overpotentials generated by high resistance at the biotic-abiotic interface. Reducing this resistance by diffusible redox mediators necessitates; (i) the formation of transient chemical interactions with the biotic and abiotic entities in question to extract and transfer electrons, (ii) a lipophilic-hydrophilic balance in the redox mediator structures to maintain permeability through membranes without getting lodged (partition coefficient) [59], and (iii) low steric bulk in order to access deeply-welled catalytic sites and to increase the diffusivity of redox mediator molecule. Lipid-soluble diffusible redox mediators hold the advantage of being able to freely diffuse across lipid membranes, whereas lipid-insoluble diffusible redox mediators typically have higher electron transfer rates [129]. Among lipid-soluble redox mediators, membrane permeability of thionine is typically higher than phenothiazine, quinone, and azo compounds [142]. Reducing the biotic-abiotic interfacial resistance by redox polymers necessitates; (i) their secure immobilization on the electrode, (ii) hydrogel formation to load a maximum of the biotic components and form chemical interactions for electron extraction, (iii) and (iv) fast electron conduction or high electric conductivity through the polymer.

Additionally, the redox species must be biocompatible and air stable for viable photobioelectrocatalytic performance. The physical color of the redox mediating species is no longer a major concern. Overlapping absorption wavelengths between photocatalysts and redox mediators instigate competition between the two for light absorbance. While this is not ideal behavior, the competition can be mitigated by tuning the photobioelectrochemical cell architecture. For instance, the layer-by-layer deposition of the redox polymer and then the photocatalyst on the electrode surface, exposes the latter to more light. Conversely, complementary absorption wavelengths between photocatalysts and redox mediators can enhance photo-harvesting. An emerging alternative route to optimize solar absorbance is the tandem application of solid-state photovoltaics, which can make efficient use of the solar spectrum (e.g., molecular dyes, colloidal quantum dots, semiconductors, etc. [196, 197], replacing photo-absorbance by the biotic component either fully or partially [19].

Poor electrical contact at biotic-abiotic interfaces during PBEC is an overarching challenge applicable to almost all bioelectrocatalytic systems. Therefore, the extensive knowledge available on redox mediation in bioelectrocatalysis can be constructively adapted to PBEC. For instance, diffusible redox mediators HNQ and thionine which have been used with microbial fuel cells of *E. coli* and *Gluconobacter oxydans* have also been utilized with cyanobacteria *Anabaena * [198–200].

### Upcoming systems and approaches

Computational studies based on density functional theory approaches are being used to predict redox potentials, D_app_, electron transfer rates at interfaces, among other parameters in order to provide comprehensive and fast libraries of redox mediating species [50, 201]. The integration of machine learning can infer logical patterns that have hitherto been incognizant in the extensive research history of redox mediators spanning nearly four decades. Computational models can also decipher the electron transfer mechanisms at complex biotic-abiotic interfaces. This burgeoning field of science will lead to incisive selection and design of redox mediating species for specific biotic-abiotic interfaces.

Genetic modification of biological components in photobioelectrochemical cells is becoming a significantly successful approach in order to forgo or elevate the performance of redox mediators [40]. Current density, which correlates to the metabolic rate of microbes, can be tweaked genetically [160]. Overcoming electron sinks and improving the electric conductivity of membranes by over-expressing genes required for incorporating more electron transfer routes across the membrane are such instances. Synthetic biology and protein engineering are utilized to over-express endogenous soluble electron mediators, redox active proteins such as cytochrome C and mtrCAB complex in photosynthetic biological entities, and incorporate natural exoelectrogenic pathways into new microbes [202–204]. Careful selection of these modifications is required, to avoid the frequently associated side-production of harmful species, unknown metabolic effects, deteriorated cell growth and unstable recombinant DNA.

## Abbreviations

AzB: Azure blue
BQ: Benzoquinone
Dapp: Apparent electron diffusion coefficient
DCBQ: Dichloro benzoquinone
DCPIP: Dichlorophenolindophenol
DMBQ: Dimethyl benzoquinone
DSSC: Dye-sensitized solar cell
E-DET: Extracellular direct electron transfer
EET: Extracellular electron transfer
EIS: Electrochemical impedance spectroscopy
E-MET: Extracellular mediated electron transfer
HNQ: Hydroxy naphthoquinone
IDA: Inter-digitated array
ITO: Indium tin oxide
LHC: Light harvesting center
MB: Methylene blue
Micro-BSC: Micro-bio solar cell
MV: Methyl viologen
NB: Nile blue
NQ: Naphthoquinone
NQS: Naphthoquinone sulfonate
NR: Neutral red
PEDOT: PSS: Poly(3,4-ethylene dioxythiophene): polystyrene sulfonate
PPBQ: P-Phenyl benzoquinone
PS: Photosystem
ROS: Reactive oxygen species
TB: Toluidine blue
TMBQ: Tetramethyl benzoquinone
TMPD: Tetramethyl-p-phenyldiamine
